# Opioid-Free Anesthesia for Upper Limb Surgery in Obese Patients as a Day Case Surgery: A Prospective Observational Study

**DOI:** 10.5812/aapm-150997

**Published:** 2024-12-16

**Authors:** Rana Ahmed Abdelghaffar, Mohamed Ahmed Hamed, Mohammed Magdy Basiony, Mohammad Fouad Algyar, Omar Sayed Fargaly, Mohamed Ahmed Shawky

**Affiliations:** 1 Department of Anesthesiology, Faculty of Medicine, Fayoum University, Faiyum Governorate, Egypt; 2 Department of Anesthesiology, Faculty of Medicine, Kafrelsheikh University, Kafr El-Sheikh Governorate, Egypt

**Keywords:** Opioid-Free Anesthesia, Upper Limb Surgery, Obese Patients, Day Case Surgery, Dexmedetomidine, Postoperative Pain

## Abstract

**Background:**

Opioid-free anesthesia (OFA) is a relatively new approach, and many studies are still needed to assess its effectiveness and compare it to opioid-based anesthesia (OBA).

**Objectives:**

This study investigated the use of OFA in obese patients undergoing upper limb surgery and compares its outcomes with those of OBA.

Methods:

This prospective randomized clinical study included 76 obese patients with a Body Mass Index (BMI) ≥ 30 kg/m² who were scheduled for upper limb surgery. Patients were randomly assigned to receive either OFA (group A, n = 38) or OBA (group B, n = 38). The OBA group was administered propofol, fentanyl, and atracurium, while the OFA group received lidocaine, propofol, atracurium, and dexmedetomidine. All patients were mechanically ventilated, and anesthesia was maintained with isoflurane and atracurium. Primary outcomes monitored included postoperative pain [Visual Analog Scale (VAS) ≥ 4] and the number of rescue doses of tramadol. Secondary outcomes included extubation time, any cardiac events, hypoxia, postoperative nausea and vomiting (PONV), intensive care unit (ICU) admission rates, and duration of hospital stay.

**Results:**

The OFA group had significantly lower extubation time, mean arterial pressure (MAP), and heart rate (HR) compared to the OBA group. Additionally, VAS scores were significantly lower at the 30-minute and 2-hour marks after extubation (P < 0.001 and P < 0.001, respectively) in patients receiving OFA. The OFA group also experienced fewer adverse effects, required fewer rescue doses of tramadol, and had shorter hospital stays.

**Conclusions:**

Opioid-free anesthesia may result in better and safer outcomes for obese patients undergoing upper limb surgeries, with fewer postoperative complications and shorter hospital stays. However, further research is needed to fully understand the potential benefits of OFA compared to OBA.

## 1. Background

The use of opioids in clinical anesthesia practice is widespread. Although opioids are effective analgesics and commonly used to manage perioperative pain, they are associated with many side effects, including respiratory depression, delirium, impaired gastrointestinal function, urinary retention, postoperative nausea and vomiting (PONV), and addiction. The most significant side effect for patients with obesity is respiratory depression (1, 2).

Obesity can lead to increased mechanical compression of the diaphragm and lungs, which may reduce functional residual capacity and total lung compliance (3). Analgesics and anesthesia, particularly opioids, exacerbate these respiratory issues by increasing the risk of hypoxia (4).

One way to avoid these side effects is by using opioid-free anesthesia (OFA). Opioid-free anesthesia has recently gained popularity and applicability as it improves pain management while eliminating the need for opioids. However, further research and studies are still needed to fully understand the mechanisms and techniques involved (5, 6).

The OFA technique is not simply a one-drug replacement for opioids, but rather a combination of drugs that work together to achieve effects similar to those of opioids. These include hypnotics, sodium channel blockers, anti-inflammatory drugs, and alpha-2 agonists (7, 8).

## 2. Objectives

In our study, we compare OFA and opioid-based anesthesia (OBA) in terms of efficacy and side effects. Our goal is to improve care quality for obese patients by enabling faster recovery with fewer side effects and complications.

This study hypothesizes that OFA will result in less postoperative pain and fewer complications.

## 3. Methods

This prospective randomized clinical study was registered at ClinicalTrials.gov under the NCT05481970 number (principal investigator: Rana Ahmed Abdelghaffar), with the registration date of 01/08/2022. The study was conducted from September 2022 through November 2023. After approval from our local ethical committee (D293), and upon receiving written informed consent, 76 patients scheduled for upper limb surgery under general anesthesia were included.

### 3.1. Inclusion Criteria

- Patients aged 18 - 60 years, of both sexes, with ASA physical status II or III, and a Body Mass Index (BMI) ≥ 30 kg/m², scheduled for upper limb surgeries (e.g., orthopedic, plastic) under general anesthesia.

### 3.2. Exclusion Criteria

- Patients with allergies to any of the drugs used in the study.

- Pregnant or lactating women.

- Individuals with a history of opioid addiction or recent opioid use.

- Patients unable to use the Visual Analog Scale (VAS).

- Patients with hepatic, cardiac, or renal diseases.

- Epileptic patients.

- Patients with cerebrovascular disease.

### 3.3. Randomization and Blinding

Patients were randomly assigned to treatment groups in a 1:1 ratio using a computer-generated random table and the closed envelope method. The trial was double-blind, with the participant, clinical care team, and assessor all blinded to the treatment allocation. Allocation concealment was achieved using a centralized web-based randomization system.

All patients underwent a preoperative clinical examination and routine preoperative laboratory investigations. Prior to surgery, patients were trained on how to use the VAS. The VAS is a pain scale represented by a 10 cm line, with endpoints denoting “no pain” on the far left and “the most intense pain” on the far right. It is used to record pain levels for individual patients and to compare pain levels among different patients (9, 10).

In the operating room (OR), we monitored and recorded baseline peripheral oxygen saturation and blood pressure readings. All patients received 1 mg of midazolam via a peripheral intravenous cannula prior to the induction of anesthesia. Preoxygenation was performed for 3 - 5 minutes.

### 3.4. Group A (n = 38): Opioid-Free Anesthesia

We used lidocaine (1.5 mg/kg), propofol (2 - 3 mg/kg), and atracurium (0.5 mg/kg) to induce general anesthesia. Dexmedetomidine (0.5 µg/kg) was administered over 10 minutes, starting 10 minutes before induction. Following tracheal intubation, the dexmedetomidine infusion was initiated at 0.6 µg/kg/h and titrated between 0.2 and 1.0 µg/kg/h, according to the heart rate (HR) and to maintain the Bispectral Index (BIS) between 40 and 60. Lidocaine was administered at 1.5 mg/kg/h. Ketamine was given as a bolus dose of 0.3 mg/kg following induction and prior to skin incision, then continued as an infusion at 0.2 mg/kg/h. Patients also received dexamethasone (8 mg i.v.) after induction.

### 3.5. Group B (n = 38): Opioid-Based Anesthesia

Patients were given propofol (2 - 3 mg/kg) and fentanyl (1 - 2 µg/kg) to induce general anesthesia, along with atracurium (0.5 mg/kg) as a muscle relaxant to facilitate intubation. Fentanyl was administered as a bolus dose of 0.5 - 1 µg/kg to maintain the BIS score between 40 and 60.

All patients were placed on mechanical ventilation with a 50% O_2_ and 50% air mixture, and end-tidal CO_2_ levels were maintained between 30 - 35 mmHg. Isoflurane and atracurium (0.1 - 0.2 mg/kg) were administered every 20 - 30 minutes to maintain anesthesia.

At the end of surgery, we used intravenous neostigmine (0.05 mg/kg) and atropine (0.01 mg/kg) to reverse the effects of muscle relaxants. Patients were extubated once they achieved a tidal volume of ≥ 5 mL/kg and SpO_2_ > 92%. Paracetamol (1 gm i.v.) and ketorolac (30 mg slow i.v. injection) were administered at the end of surgery, before emergence, in both groups.

In the recovery room, patients were monitored for postoperative pain and asked to assess their pain level using the VAS method. They left the post-anesthesia care unit (PACU) with an Aldrete score of more than 9 (11). Any patient with a VAS score ≥ 4 received tramadol 1 mg/kg i.v., with a maximum dose of 600 mg/day.

### 3.6. Primary Outcome

Episodes of postoperative pain with a VAS score ≥ 4 were monitored and recorded starting from PACU (30 minutes after extubation, then at 2, 6, 12, and 24 hours).

### 3.7. Secondary Outcomes

- Rescue doses of tramadol (time, number, and side effects of the drug) were also recorded.

- Hypoxia (SpO_2_ level of less than 95%) with the need for oxygen supplementation, monitored in PACU and then every 2 hours for 24 hours.

- Extubation time (from the end of surgery to extubation).

- Intraoperative cardiac events (bradycardia: HR ≤ 50 bpm, hypotension: mABP ≤ 60 mmHg, hypertension: mABP ≥ 90 mmHg).

- Postoperative nausea and vomiting (number of attacks from extubation to 24 hours and need for rescue antiemetic medication).

- Intensive care unit (ICU) admission rates.

- Hospital stay duration.

### 3.8. Statistical Analysis and Sample Size Estimation

The estimated sample size was 76 (38 per group). The sample size was calculated using G*Power software version 3.1.9.6 with a power of 80%, a 5% probability of type I error, and an effect size of 0.67. The effect size was estimated to detect a difference of one point on the VAS score between the two groups, with a standard deviation (SD) of 1.5, based on the results of similar studies examining VAS scores (12). No adjustment for the sample size was made for interim analysis or potential dropouts, as there was no intention of performing interim analysis and the follow-up duration was only 24 hours, which provided a limited probability for dropouts.

Statistical analysis was performed using SPSS version 28 (IBM Co., Armonk, NY, USA). Quantitative parametric data were presented as the mean and SD and analyzed using the independent samples Student’s *t*-test. Quantitative non-parametric data were presented as the median and interquartile range (IQR) and analyzed using the Mann–Whitney test.

Categorical data were presented as frequencies and percentages and analyzed using the chi-square test or Fisher’s exact test when appropriate.

Relative risk (RR) was calculated to assess the probability of different events occurring in the OFA group versus the OBA group.

A two-tailed P-value < 0.05 was considered statistically significant.

## 4. Results

**Figure 1.**
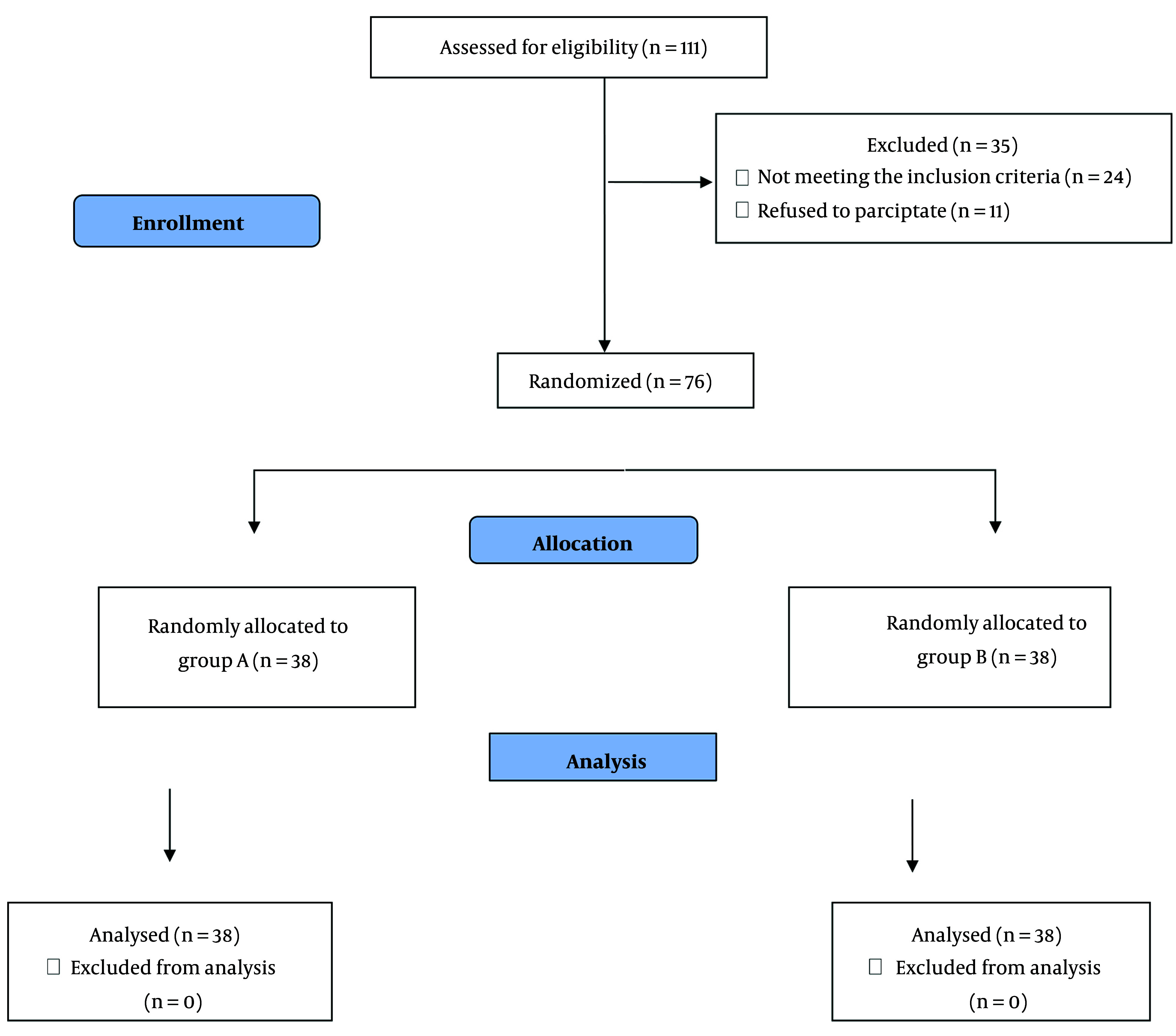
CONSORT flow diagram of the study population

One hundred eleven patients were assessed for eligibility; of those, 76 patients completed the study and were randomized (38 patients in each group). Their data were included in the final analysis. Thirty-five patients were excluded from the study due to not meeting the inclusion criteria (24 patients) and patient refusals (11 patients). The number of exclusions did not affect the study (Figure 1). 

There was no statistically significant difference between the groups regarding demographic characteristics, including age, sex distribution, BMI, and ASA physical status, ensuring the homogeneity of both groups. Both groups were comparable regarding the duration of the surgery. However, compared to patients who received OBA (group B), the extubation time for patients who received OFA (group A) was significantly lower (mean of 8.89 ± 1.72 vs 12.58 ± 2.31 minutes, respectively; P < 0.001) (Table 1). 

**Table 1.** Demographic Data and Surgery Characteristics of the Study Groups ^a^

Variables	Group A (n = 38)	Group B (n = 38)	P-Value
**Age (y)**	38.58 ± 8.97	37.89 ± 7.5	0.719
**Sex**			0.818
Male	19 (50)	20 (52.6)	
Female	19 (50)	18 (47.4)	
**BMI (kg/m** ^**2**^ **)**	35.92 ± 2.73	36.84 ± 2.99	0.165
**ASA physical status**			0.237
II	33 (86.8)	29 (76.3)	
III	5 (13.2)	9 (23.7)	
**Duration of surgery (min)**	85.53 ± 19.06	81.58 ± 17.4	0.349
**Extubation time (min)**	8.89 ± 1.72	12.58 ± 2.31	< 0.001 ^b^

Abbreviations: BMI, Body Mass Index, ASA, American Society of Anesthesiologists.

^a^ Values are expressed as mean ± SD or No. (%).

^b^ Statistically significant.

At the start of the study, HR was comparable between the two groups (P = 0.168). However, we observed a statistically significant drop in HR in Group A compared to Group B at 15, 30, 60, and 90 minutes post-induction, with this significant drop continuing after 2 and 4 hours in the PACU (P < 0.05).

Both groups had comparable mean arterial pressure (MAP) at the start of the study. However, at 15, 30, 60, and 90 minutes post-induction, MAP was substantially lower in group A than in group B (P < 0.05). Similarly, at 20 and 24 hours in the PACU, MAP was significantly lower in group A than in group B (P < 0.001 and P = 0.01, respectively).

Postoperative pain was evaluated using the VAS, which showed a statistically significant difference at 30 minutes and 2 hours after extubation (P < 0.001 and P = 0.001, respectively), with pain levels being lower in patients who received OFA (group A) compared to those who received OBA (group B). However, the difference was not significant between 6 and 24 hours after extubation. The percentage of patients reporting a VAS score ≥ 4 showed a statistically significant difference at 30 minutes and 2 hours after extubation (P = 0.012 and P = 0.001, respectively). At 30 minutes after extubation, 0% of patients in the OFA group (group A) reported a VAS score ≥ 4, compared to 18.4% in the OBA group (group B). At 2 hours after extubation, 34.2% of patients in the OFA group (group A) reported a VAS score ≥ 4, compared to 71.1% in the OBA group (group B) (Table 2). 

**Table 2.** Visual Analog Scale Scores of the Studied Groups ^a^

After Extubation	Group A (n = 38)	Group B (n = 38)	P-Value	Effect Size [r or Difference in Proprtions (95% CI)]
**VAS score**				
30 min	2 (2 - 3)	3 (2 - 3)	< 0.001 ^b^	0.45 ^c^
2 h	3 (3 - 4.25)	5 (3 - 6)	0.001 ^b^	0.38 ^d^
6 h	4 (3 - 5)	4 (3 - 5)	0.576	0.06
12 h	3 (3 - 4)	3 (3 - 5)	0.495	0.08
24 h	2 (2 - 3)	2 (2 - 3)	0.345	0.11
**VAS score ≥ 4**				
30 min	0 (0.0)	7 (18.4)	0.012 ^b^	0.18 (0.06, 0.31)
2 h	13 (34.2)	27 (71.1)	0.001 ^b^	0.37 (0.16, 0.58)
6 h	23 (60.5)	28 (73.7)	0.222	0.13 (-0.08, 0.34)
12 h	18 (47.4)	18 (47.4)	> 0.999	0.0 (-0.22, 0.22)
24 h	0 (0.0)	0 (0.0)	-	-

Abbreviation: VAS: Visual Analogue Scale.

^a^ Values are expressed as median (IQR) or No. (%).

^b^ Statistically significant at P-value < 0.05.

^c^ Medium effect size.

^d^ Large effect size.

Table 3 shows that patients in the OFA group were at significantly lower risk of developing postoperative adverse effects than those in the OBA group (31.6% vs 63.2%, respectively; P = 0.006), with a RR of 0.5. Nausea was the most frequently observed adverse effect, with a significantly lower risk in group A compared to group B (21.1% vs 52.6%, respectively; P = 0.004, RR = 0.4). Similarly, vomiting occurred significantly less often in group A than in group B (0% vs 44.7%, respectively; P < 0.001, RR = 0.03). Moreover, the number of PONV PONV attacks and the use of antiemetics were lower in group A compared to group B (P = 0.007 and P = 0.004, respectively). Notably, the rates of sedation and hypoxia were not significantly different between the two groups.

**Table 3.** Adverse Effects of the Studied Groups ^a^

Variables	Group A (n = 38)	Group B (n = 38)	P-Value	RR (95%CI)
**Adverse effects**				
No	26 (68.4)	14 (36.8)	0.006 ^b^	0.5 (0.3 - 0.85)
Yes	12 (31.6)	24 (63.2)		
Sedation	4 (10.5)	1 (2.6)	0.358	4 (0.47 - 34.16)
Hypoxia	2 (5.3)	8 (21.1)	0.086	0.25 (0.06 - 1.1)
**PONV**				
Nausea	8 (21.1)	20 (52.6)	0.004 ^b^	0.4 (0.2 - 0.8)
Vomiting	0 (0)	17 (44.7)	< 0.001 ^b^	0.03 (0.002 - 0.46)
**Number of attacks, median (IQR)**	0 (0 - 0)	1 (0 - 1)	0.007 ^b^	-
**Need for antiemetics**	8 (21.1)	20 (52.6)	0.004 ^b^	0.4 (0.2 - 0.8)

Abbreviations: PONV, postoperative nausea and vomiting; RR, relative risk; CI, confidence interval.

^a^ Values are expressed as No. (%) or unless otherwise indicated.

^b^ Statistically significant at P-value < 0.05.

Regarding hospital stay, it was significantly shorter in group A compared to group B (P < 0.001), while the ICU admission rate did not differ significantly between the two groups.

## 5. Discussion

In this study, we demonstrated the use of OFA techniques in obese patients undergoing upper limb surgeries (orthopedic, plastic, etc.) under general anesthesia. Our study included 76 patients, distributed into two groups, with 38 patients in each group. Patients who underwent OFA had shorter extubation times, lower MAP, less postoperative pain, fewer rescue doses of tramadol, and a lower risk of developing postoperative adverse effects compared to those who underwent OBA. They also had significantly shorter hospital stays, although ICU admission rates showed no significant difference between the two groups.

In our study, the two groups were similar in terms of demographic characteristics, including age, sex distribution, BMI, and ASA physical status, ensuring the homogeneity of the groups.

Furthermore, both groups had similar surgery durations, but extubation time was substantially shorter in the OFA (group A) compared to the OBA (group B). A study by Guinot et al. on cardiac surgery reported that extubation time was significantly shorter in the OFA group than in the OBA group (13). Another study by Aguerreche et al. also found that the OFA group had a shorter extubation time compared to the OBA group (14). In addition, a scoping review by Connor et al. found that opioids are clinically proven to prolong the time to extubation (15). In contrast, a study on lung cancer patients by An et al. reported that the recovery and extubation times in the OFA group were significantly longer than those in the OBA group (16).

In this study, both groups had similar HRs and MAP at the start. However, HR was lower in the OFA group at 15, 30, 60, and 90 minutes post-induction, as well as at 2 and 4 hours in the PACU. In agreement with the results of this study, a study by Elsaye et al. reported a decrease in HR and MAP in the OFA group compared to the OBA group from 15 minutes after induction to 15 minutes postoperatively (17). However, Mulier et al. found no difference in HR and MAP between the OFA and OBA groups during laparoscopy, contradicting the previous study. This discrepancy may be due to sufentanil's myocardial stability and potency over fentanyl (18).

Regarding the VAS score of the studied groups, postoperative pain levels in the OFA group were lower than in the OBA group at 30 minutes and 2 hours after extubation, but not at 6 to 24 hours after extubation. The percentage of patients with a VAS pain level ≥ 4 in the OFA group was also significantly lower than in the OBA group at 30 minutes and 2 hours after extubation. However, a study by Elsaye et al. reported that the VAS score was significantly lower in the OFA group than in the OBA group at all postoperative time points, from 0 hours to 24 hours (17). Moreover, Shalaby et al. reported substantial differences in VAS scores between the dexmedetomidine and fentanyl groups at 20, 60 minutes, and 6 hours postoperatively, with lower VAS scores in the dexmedetomidine group (19). In contrast, Choi et al. noted no significant difference in VAS scores for postoperative pain between the dexmedetomidine, fentanyl, and remifentanil groups, suggesting that fentanyl and remifentanil have stronger analgesic effects than dexmedetomidine when used alone for OFA (20).

Furthermore, compared to OBA, OFA was reported to have a much lower risk of postoperative adverse effects such as nausea, vomiting, PONV attacks, and the need for antiemetics. However, the sedation and hypoxia rates were the same in both groups. Another study by Choi et al. reported that OFA is a safe and effective technique for providing intraoperative hemodynamic stability and postoperative analgesia with fewer associated adverse effects than OBA (20). However, a meta-analysis by Salome et al. found no clinically significant benefits of OFA over OBA in terms of pain and opioid use (21). Nevertheless, OFA was associated with a reduction in PONV. More data is needed on the safe use of OFA, and caution is necessary in its development. Additionally, a trial by Beloeil et al. found that balanced OFA with dexmedetomidine was not associated with fewer postoperative opioid-related adverse events than remifentanil. In fact, it was associated with a greater incidence of serious adverse events, especially hypoxemia and bradycardia (22).

Regarding rescue analgesic requirements, in our study, patients in the OFA group required significantly fewer rescue doses of tramadol for a significantly longer time after surgery than patients in the OBA group. This finding is consistent with recent studies, including one by Bhardwaj et al., which reported that significantly fewer patients in the opioid-free group required rescue analgesia (4).

In addition, a study by Bakan et al. found that OFA was associated with significantly lower pain scores, and a reduced need for rescue analgesics and ondansetron (6).

In our study, we found that the hospital stay was significantly shorter in the OFA group than in the OBA group, while the ICU admission rate did not differ significantly between the two groups. A study by Guinot et al. on cardiac surgery reported that ICU stays were significantly shorter in the OFA group than in the OBA group (13). However, a study by Kharasch and Clark found no differences between groups regarding ICU admission and length of stay (23). A single-center study by Martin et al. reported that the transition to OFA for laparoscopic appendectomy led to a decrease in the mean hospital length of stay from 2.9 to 1.4 days, saving more than 500 hospital patient days per year (24).

### 5.1. Limitations

All participants in this clinical trial were from Egypt, limiting the generalizability of the data to other races. Additionally, VAS scores were assessed only during the 24-hour postoperative period, and the long-term implications of OFA in different surgical populations were not explored. Furthermore, the study had a small sample size and limited follow-up.

### 5.2. Conclusions

This study highlights the potential benefits of OFA over OBA. Patients in the OFA group experienced shorter extubation times, reduced postoperative pain, fewer rescue doses of tramadol, and a lower risk of developing postoperative adverse effects. Additionally, the OFA group had shorter hospital stays. These findings underscore the potential of OFA to improve the safety and overall experience of obese patients undergoing surgery.

## Data Availability

The datasets used and analyzed during the current study are available from the corresponding author upon reasonable request.

## Clinical Trial Registration Code:

NCT05481970.
